# Potential health effects of electronic cigarettes: A systematic review of case reports

**DOI:** 10.1016/j.pmedr.2016.06.002

**Published:** 2016-06-10

**Authors:** My Hua, Prue Talbot

**Affiliations:** a Environmental Toxicology Graduate Program, University of California, Riverside, Riverside, CA, United States; b Department of Cell Biology and Neuroscience, University of California, Riverside, Riverside, CA, United States

**Keywords:** Electronic cigarettes, Tobacco products, Adverse health effects, Nicotine, Poisoning, Explosions

## Abstract

The health risks associated with electronic cigarettes (ECs) are largely unknown. The purpose of this systematic review was to evaluate published case reports that deal with health effects attributed to EC use. An Internet search was conducted to identify case reports dealing with the effects of EC use on health. Twenty-six case reports representing 27 individuals (one study contained reports for two individuals) were published between April 2012 and January 2016, and these were grouped into categories of effect according to their health outcomes.

Of the 27 individuals, 25 had negative effects subsequent to use or exposure to ECs and their refill fluids, while two reported improvement in chronic immune and gastrointestinal conditions. Three categories of negative health effects were identified: systemic effects, nicotine poisoning, and mechanical injury. Thirteen cases reported EC effects on different systems including: respiratory (6), gastrointestinal or developing intestine of an infant (3), cardiovascular (2), neurological (1), and immune (1). Twelve cases involved nicotine poisoning resulting from accidental (*N* = 3), misuse/abuse (*N* = 1), or suicidal/intentional ingestion (*N* = 8); four of these involved children and three resulted in adult fatalities. Two cases reported mechanical injury caused by an EC battery explosion. Most case reports show that the health of children and adults can be negatively affected by EC products and that if death does not occur, negative effects can be reversed. Data further indicate that EC use can cause negative health effects in previously healthy individuals and exacerbate pre-existing conditions.

## Highlights

• Diverse systemic effects were associated with EC use in case reports.
• Three adult deaths from nicotine poisoning were linked to EC use.
• EC use can potentially trigger inflammation-related conditions in EC users.
• Healthy individuals and those with pre-existing conditions can be adversely affected by EC use.

## 1 Introduction

Electronic cigarettes (ECs) are battery-operated devices that heat a liquid containing propylene glycol and/or glycerin, nicotine, and flavorings to produce an aerosol which users inhale (Trtchounian and Talbot, 2011, Grana et al., 2014). In spite of their rapid rise in popularity and worldwide sales, the effects of EC use on short and long-term human health are poorly understood. Because EC aerosol contains fewer chemicals than conventional tobacco smoke, EC are often considered a safe alternative to cigarettes. However, EC are not without health risks.

Information linking EC use to health effects has been gained mainly from short-term *in vitro* studies with cultured cells and *in vivo* experiments with human subjects (Pisinger and Døssing, 2014). *In vitro* studies have shown that cytotoxic effects vary among EC refill fluids (Bahl et al., 2012), some flavor chemicals (such as cinnamaldehyde) have toxicity at the concentrations used in EC (Behar et al., 2014a, Behar et al., 2014b, Lerner et al., 2015), and stem cells are more sensitive than differentiated adult lung cells to EC products (Bahl et al., 2012, Behar et al., 2014a). Recent studies have further shown that EC aerosols induced DNA strand breaks and reduced cell survival *in vitro* (Yu et al., 2016). EC aerosols also reduced endothelial barrier function in cultured lung microvascular endothelial cells and increased inflammation and oxidative stress in mice (Schweitzer et al., 2015, Sussan et al., 2015). Most *in vivo* studies have involved experiments with human subjects, and these were recently reviewed (Pisinger and Døssing, 2014). An infodemiological study of online forums found 405 different health-related effects (78 positive, 326 negative, 1 neutral) attributed by users to EC (Hua et al., 2013). Of recent concern is the finding that a high percentage of EC refill fluids contain toxicants such as diacetyl and diethylene glycol (Westenberger, 2009, Varlet et al., 2015, Allen et al., 2015), and EC aerosols contain formaldehyde-hemiacetals, ultrafine particles, and metals (Jensen et al., 2015, Williams et al., 2013, Williams et al., 2015). The EC industry is making hundreds of new EC models and thousands of flavors of e-liquids or refill fluids available to consumers (Zhu et al., 2014, Tierney et al., 2015). Although many of these flavors are considered safe for ingestion by the Flavor Extracts Manufacturers Association (FEMA), their inhalation safety has not been established by FEMA (Hallagan, 2015).

Additional information on EC health effects can be gained from case reports on EC users who present to physicians with symptoms attributed to EC use or exposure. The number of case reports that appears in the peer-reviewed literature has reached a critical mass and is currently worth mining to gain further insight into the positive and negative health effects appearing in EC users. The purpose of this study was to systemically collate and analyze the existing case reports linking EC use to health outcomes and to identify categories of health effects related to EC use.

## 2 Methods

**Fig. 1:**
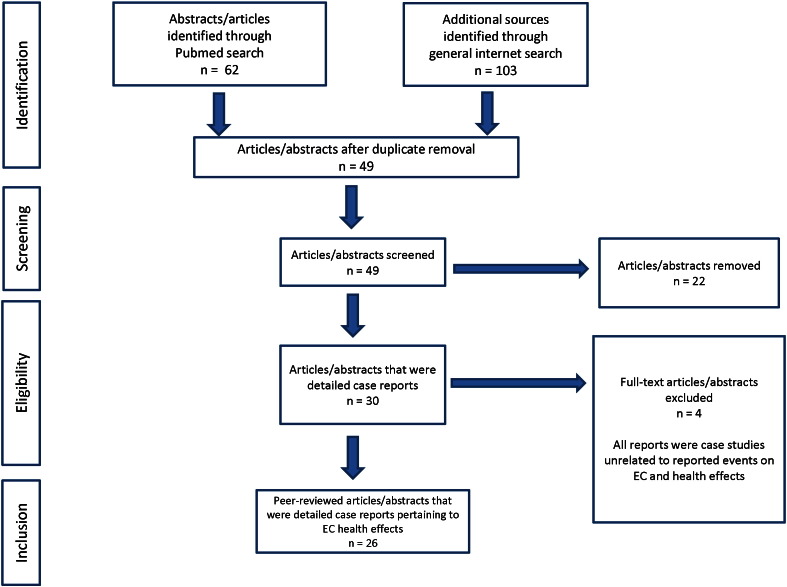
Flowchart of the procedure used to collect case reports that are included in the systematic review.

This review follows the PRISMA (Preferred Reporting Items for Systematic Reviews and Meta-analysis) guidelines for conducting systematic reviews. The flowchart for the systematic review is represented in Fig. 1.

### 2.1 Search strategy

An Internet search using the keywords or phrases: “electronic cigarette”, “e-cigarette case reports”, “e-cigarette case studies”, and “e-cigarette nicotine poisoning case reports” was carried out in PUBMED, MEDLINE, and general internet search engines. Additional searches with the keywords followed by year were used (*i.e.* “e-cigarette case report 2012”). References in case reports or other related peer-reviewed literature also contributed to gathering literature.

### 2.2 Inclusion criteria

Case reports and reviewed abstracts dealing with health effects attributed to EC use or exposure were included. At a minimum, the cases reporting systemic effects needed to: (1) clearly discuss the patient's symptoms upon presentation and (2) be reported and diagnosed by healthcare professionals.

Similarly, cases reporting nicotine poisonings or mechanical injuries needed to provide information concerning the context of EC use and/or summarize health impacts on the patient documented by a healthcare professional.

### 2.3 Exclusion criteria

Case reports or abstracts that reported on EC use or EC products without direct correlation to health effects were excluded from the study. Reports that dealt with health effects caused by liquid nicotine unassociated with EC were not included.

### 2.4 Identification and selection of case reports

The search strategy identified 49 articles that attributed health effects to EC use or EC products. Twenty-two of these consisted of original case reports and abstracts that matched search criteria for articles dealing with health effects attributed to EC use or exposure. Nineteen were in the peer-reviewed literature and five were reviewed abstracts (one of these presented two individual cases). The authors agreed on the inclusion criteria for case reports and reviewed conference abstracts that contained sufficient details on health effects related to EC for inclusion in the Results section. The general search yielded 27 articles and news reports that were not included in the evaluation; however, five of these which relate to death and serious illness as well as two regarding perception of EC use during pregnancy are included in the Discussion section. In addition, examination of reference lists in case reports and articles (Ruokolainen et al., 2015, Kim and Baum, 2015) that cited case reports yielded four reports for inclusion (Eberlein et al., 2014, Schipper et al., 2014, Bartschat et al., 2015, Gupta et al., 2014). These literature references also yielded one additional case that reported that adverse health effects associated with liquid nicotine not found in the online search (Kivrak et al., 2014). This case was confirmed to be linked to EC use by correspondence with the original authors (Kivrak et al., 2014).

## 3 Results

### 3.1 Overview of case reports

Of 26 case reports for 27 individuals identified in the Internet search, 25 individuals experienced negative health effects and 2 reported positive or improved outcomes attributed to EC usage (Table 1). The case reports came from 10 countries and included pediatric and adult populations ranging in age from newborn (1 day old) to 70 years old. Most users who experienced health effects were adults (male *N* = 15; female *N* = 7) and five were children. A few case reports dealt with EC users who were former smokers (*N* = 3) or quit smoking for one month or more (*N* = 3). Three deaths were associated with EC use. The case report cohort included 13 individuals with pre-existing health conditions (eight systemic; five mental health disorders). Among the 13 individuals, 11 experienced negative health effects. Four experienced health effects related to worsening of a preexisting condition.

Four of six patients who used EC for smoking cessation experienced negative effects. Of these, three patients were dual users of EC and cigarettes, and two experienced negative effects, while one had positive effects.

Three categories of health effects attributed to EC use could be identified in the case reports and abstracts. Systemic health effects (*N* = 13) were associated with conditions relating to body systems/organs. Nicotine poisonings (*N* = 12) were associated with ingestion or internalization of liquid nicotine, and varying symptoms of nicotine poisoning were reported for adults and pediatrics. Mechanical injury (*N* = 2) included adverse effects resulting from EC explosions. The median age for systemic events was 42. For nicotine poisonings the median age was 23. The patients injured by mechanical explosion were both male, aged 18 and 30. All reviewed case reports indicated no conflicts of interest; for reviewed abstracts, no statements on conflicts of interest were given. Each of these categories will be discussed in order of reporting prevalence.

#### 3.1.1 Respiratory system

Six case reports involved the respiratory system. The specific diagnoses were two cases of exogenous lipoid pneumonia (McCauley et al., 2012, Modi et al., 2015), and one case of each of the following: bronchiolitis (Hureaux et al., 2014), acute eosinophilic pneumonia (Thota and Latham, 2014), pneumonia with bilateral pleural effusions (Moore et al., 2015), and inhalation injury and suspected acute hypersensitivity pneumonitis (Atkins and Drescher, 2015). The patient who experienced acute eosinophilic pneumonia was described as previously healthy. One patient with lipoid pneumonia had adverse respiratory health effects that coincided with 7 months of EC use. The patient with bronchiolitis had previously been treated for pulmonary adenocarcinoma, and the patient with pneumonia with bilateral pleural effusions had a history of hypertension.

Upon presentation, the patients with negative effects were experiencing symptoms such as shortness of breath and cough. Although each diagnosis was different for five of six patients, the six patients with negative effects were all diagnosed with inflammation of the respiratory system. For the individuals with bronchiolitis, acute eosinophilic pneumonia, and pneumonia with bilateral pleural effusions, the onset of symptoms occurred within 3 days–7 days of EC use. In the two cases of lipoid pneumonia, the patients experienced an onset of respiratory symptoms 7 months and 3 months after starting EC use. The patient who experienced inhalation injury and suspected acute hypersensitivity pneumonitis had a history of previous medical admittance related to respiratory concerns and he had used EC before his prior medical admittance.

Four of the patients' conditions improved and they had no further symptoms after seeking medical care and abstaining from EC use. The conditions of the two patients diagnosed with acute eosinophilic pneumonia and pneumonia with bilateral pleural effusions also improved, however, it was not reported if they abstained from EC use in addition to their medical treatment.

Details concerning refill fluid flavors were provided in two of the six respiratory cases. The patient diagnosed with bronchiolitis was using two different brands of EC liquids with tobacco flavoring that had nicotine concentrations of 19 mg/ml, while the patient suffering from inhalation injury and suspected acute hypersensitivity pneumonitis used tobacco and an unspecified “sweet flavoring containing diacetyl”.

Little information on EC usage was given. The patient with bronchiolitis used his EC 25–26 times per day, and in each session, he reported taking approximately 5–6 puffs. In addition to EC use, this patient smoked 20 cigarettes a day. The patient diagnosed with pneumonia with bilateral pleural effusions estimated that he puffed hundreds of times/day for 3 days.

#### 3.1.2 Gastrointestinal system

Three case reports involved the gastrointestinal system. The specific diagnoses were relapsed ulcerative colitis (UC) (Camus et al., 2014), clinical remission of UC (Lee et al., 2013), and necrotizing enterocolitis in the developing gut of an infant (Gillen and Saltzman, 2014). Both adult patients (a male and female) had a history of UC and were previous smokers.

Upon presentation, the patients with negative effects experienced different gastrointestinal symptoms. The patient with relapsed UC experienced bloody diarrhea. The infant who had necrotizing enterocolitis experienced intestinal bleeding. The patient with clinical remission of UC had experienced bloody bowel movements with severe incontinence before EC usage.

For the individuals with relapsed UC and remission of UC, the onset of symptoms occurred 3 months after the patient quit smoking and 4 weeks after smoking cessation and initiation of EC use, respectively. In the case of the infant with necrotizing enterocolitis who was exposed to EC *in utero*, symptoms began shortly after birth.

The patient with relapsed UC experienced improvement in her condition after she stopped EC use and resumed smoking. The infant experiencing necrotizing enterocolitis required multiple surgeries, but follow-up reports indicated he recovered and developed normally after the surgeries. The patient who experienced UC remission after EC use continued EC usage and smoking cessation. However, there was no additional follow-up on this patient. The patient who experienced relapsed UC was using both EC refill fluid with 30 mg of nicotine/ml daily and a cartridge with 16 mg of nicotine/ml every 5 days. For the patient who experienced remission in UC, no nicotine concentration was noted; however, he was taking an average of 105 puffs/day from his EC. The mother whose infant was born with necrotizing enterocolitis used her EC approximately 30–50 times/day for a duration of less than 5 min/session, and, during the time of active labor she was using her EC for approximately 50–70 times/day. It was also estimated that she had taken in 0.8–1 mg of nicotine daily and this increased to 1–1.4 mg before labor.

#### 3.1.3 Cardiovascular system

The two cases involving the cardiovascular system were diagnosed as paroxysmal atrial fibrillation (PAF) and acute myocardial infarction (Monroy et al., 2012, Kivrak et al., 2014). The patient who experienced PAF was an elderly female who was a dual user of EC and conventional cigarettes. She had a history of high blood pressure and was receiving care at a nursing facility. The patient diagnosed with acute myocardial infarction was a young previously healthy male (24 years old). Both patients had used EC that contained nicotine, and the previously healthy male reported using refill fluid that contained 16 mg of nicotine/ml. No details on usage patterns or flavors were provided for either patient.

For the elderly patient, a clear initiation for onset of symptoms was not described, but for the individual who experienced acute myocardial infarction, symptoms began during EC use. The patient who experienced PAF developed asymptomatic acute episodes of atrial fibrillation with rapid ventricular response. The patient who experienced acute myocardial infarction presented with chest pain.

The patient experiencing PAF was transferred to a cardiac catheterization laboratory and was administered medication intravenously. Both patients with cardiovascular effects associated with EC use had improved symptoms after appropriate medical care and monitoring of their conditions. The patient with PAF was advised to abstain from EC use and her condition improved to normal. The young male who experienced acute myocardial infarction also improved to normal after medical treatment and was discharged.

#### 3.1.4 Neurological system

One individual experienced reversible cerebral vasoconstriction syndrome (RCVS) (Vannier et al., 2015). This patient had a smoking history of 60 cigarettes/day for 20 years, but did not have a prior medical history. The individual presented to an emergency room with a 7 day history of headaches and two seizures. The patient was a dual user of EC and conventional cigarettes. He began experiencing an onset of headaches 2 days after starting EC use and smoking 20 cigarettes/day. At days 3 and 4, the patient experienced severe thunderclap headaches. The EC used by this individual had a nicotine concentration of 12 mg/ml; however, details were not provided on frequency of EC usage. The patient received medical care and was advised to abstain from EC use. The patient continued to smoke 10–15 cigarettes/day, and his headaches resolved after 3 days. A 1 month follow-up with MRI confirmed RCVS with resolving stenosis and overall improvement in his condition after stopping EC use completely.

#### 3.1.5 Immune system

One case reported a positive health effect associated with EC use for a male patient with previous history of idiopathic neutrophilia (Farsalinos and Romagna, 2013). This case report followed the patient for 7 years (from 2005 to 2012) after his initial diagnosis. The patient was a smoker who previously attempted unsuccessful smoking cessation. He had been treated for high cholesterol since 2003. In 2012, the patient started using EC for smoking cessation and was successful within 10 days. After 6 months of EC use and smoking cessation, the patient experienced a reversal of idiopathic neutrophilia at which time his condition returned to baseline/normal count.

#### 3.1.6 Mechanical injury

Two cases of mechanical injury have been reported in the peer reviewed literature (Jablow and Sexton, 2015, Rogér et al., 2016). The first involved an adult male who received severe right leg burns as a result of a spontaneous explosion of his EC battery. An image of the device was provided in the case report; however, no further details concerning the EC brand, model or battery were available.

The second case involved an adult male who suffered oral injuries when an EC he was using exploded in his mouth. The patient suffered oral and abdominal burns, oral lacerations, tooth fractures, and tooth avulsions. The patient received follow-up dental care to evaluate his oral trauma. No further details were provided concerning the EC device brand, model or battery.

#### 3.1.7 Nicotine poisonings associated with EC use and routes of exposure

Eleven case reports for 12 individuals (4 children and 8 adults) document nicotine poisonings. These reports describe both accidental and intentional/suicide attempts associated with EC. Cases reporting nicotine poisonings for both accidental and intentional/suicidal attempts involved five different routes of administration including: (1) ingestion of EC liquid nicotine (*N* = 11), (2) intravenous injection (*N* = 2), (3) dermal exposure (*N* = 1), (4) use of EC with other drug substances and/or additional sources of nicotine (*N* = 2), and (5) combined routes of administration (such as ingestion and intravenous injection to EC liquid nicotine) (*N* = 2).

#### 3.1.8 Accidental nicotine poisonings associated with EC

Three cases of accidental nicotine poisonings associated with EC involved children (Bassett et al., 2014, Gupta et al., 2014, Gill et al., 2015). The children in the reports ingested liquid nicotine which was reported in two cases to be wintergreen and grape flavored. These children experienced varying effects from nicotine poisoning that were either mild (two cases) or serious (one case). All children had symptoms of vomiting. For mild cases, one child experienced vomiting after ingestion of EC refill fluid, while the other child presented with irritability and behavioral changes. In the one severe case of nicotine poisoning, the infant experienced tachycardia, respiratory changes, and ataxia. In all cases the children recovered from nicotine poisoning following medical treatment.

#### 3.1.9 Intentional misuse/suicidal attempts associated with EC

Eight case reports involving nine individuals (8 adults and 1 adolescent) are categorized as intentional or suicide attempts related to EC (Cervellin et al., 2013, Christensen et al., 2013, Valento, 2013, Eberlein et al., 2014, Schipper et al., 2014, Thornton et al., 2014; Bartschat et al., 2015, Chen et al., 2015). One case reported accidental overdose by intravenous injection of EC liquid combined with use of other recreational drugs. Six other individuals attempted suicide by internalization of EC liquid using varying routes of exposure. Nicotine concentrations in refill fluids varied from 18 to 100 mg/ml.

Of eight attempted suicides, three deaths have been reported (Thornton et al., 2014, Bartschat et al., 2015, Chen et al., 2015). These cases reported that EC liquid nicotine was primarily ingested or intravenously injected at above lethal doses (2000 ng/ml in Thornton et al., 2014, 3950 mg in gastric contents, Bartschat et al., 2015, and 1000 ng/ml in Chen et al., 2015). Five of the eight individuals who attempted suicide had a history of mental illness, including the three individuals who died.

#### 3.1.10 Nicotine usage reported in case reports

Nicotine concentrations were provided in six systemic and six poisonings cases reports. For systematic events, the average nicotine concentration in EC products was 14.4 mg/ml ± 3.9 with a range of 9–19 mg/ml. For nicotine poisonings, the average nicotine concentration available to EC users was 29.1 mg/ml ± 19.4 with a range of 18–100 mg/ml.

## 4 Discussion

**Fig. 2:**
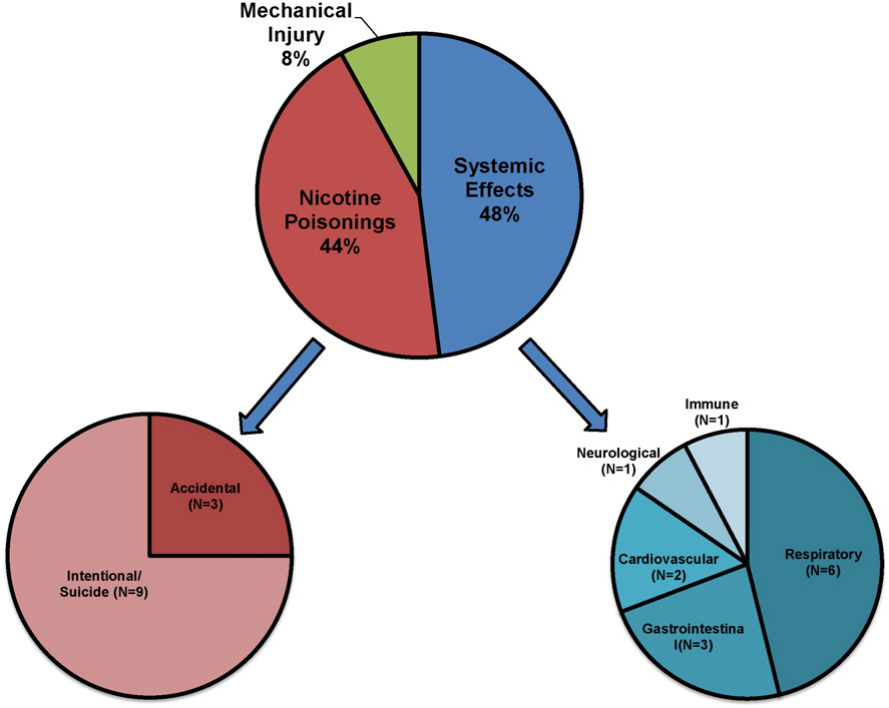
Summary of all case reports and categories of effects.

The number of peer reviewed case reports dealing with EC is now sufficiently large to provide insight into the negative and positive health effects that EC can have. The types of health effects attributed to EC are summarized in Fig. 2. Most case reports involved system effects and poisonings. System effects were diverse and some involved an immune response including inflammation that occurred in the respiratory and gastrointestinal systems (McCauley et al., 2012, Hureaux et al., 2014, Thota and Latham, 2014, Moore et al., 2015, Atkins and Drescher, 2015, Modi et al., 2015, Camus et al., 2014, Gillen and Saltzman, 2014, Thota and Latham, 2014). The systems with most reports were respiratory (*N* = 6), gastrointestinal (*N* = 3), and cardiovascular (*N* = 2), which is in agreement with our prior infodemiological report dealing with adverse health effects reported by EC users in online forums (Hua et al., 2013). Certain conditions associated with EC use, such as acute myocardial infarction in a previously healthy young male, RCVS, and necrotizing enterocolitis, are medically uncommon. In addition to the two peer-reviewed cases of lipoid pneumonia associated with EC usage, two news outlets reported separate cases in Spain and the UK (The Local, 2014, The Journal, 2011). One patient developed lipoid pneumonia after he continuously used an EC while recovering in a hospital in Spain, and another report links the death of a UK man to lipoid pneumonia with EC usage.

While there were 12 peer-reviewed case reports linking nicotine poisonings to EC products, the actual number of such poisonings is rapidly increasing according to Poison Control Center reports and exceeded 3000 in 2014 (Chatham-Stephens et al., 2014). The potential for accidental poisoning is increased by readily available EC products with high concentrations of nicotine, flavors that are attractive to children, and the lack of accurate labeling on some EC products that contain nicotine (Trehy et al., 2011; Davis et al., 2015a, Davis et al., 2015b). Poisoning of children could be reduced by child-proofing EC refill fluid products, which is already done by some manufacturers and will be required of all EC products containing nicotine once the Child Nicotine Poisoning Prevention Act goes into effect (Civic Impulse, 2016).

In addition to the three deaths in Table 1, seven deaths have been linked to EC products in the FDA-CTP database (Durmowicz, 2014) and major news outlets. These include adults and young children from the US, UK, Europe and Israel (Mohney, 2014, BBC News, 2014, Kloosterman, 2013, The Journal, 2011, The DailyMail, 2015). Four adults died from nicotine poisoning due to suicide in the US and Europe (BBC News, 2014, Thornton et al., 2014, Bartschat et al., 2015, The DailyMail, 2015); one man from the UK died from EC mechanical injury due to an explosion that ignited his oxygen equipment (BBC, 2014); and one died from systemic effects attributed to lipoid pneumonia in the UK (The Journal, 2011). Two children from the US (Mohney, 2014) and one child from Israel died (Kloosterman, 2013) due to nicotine poisoning from ingestion of EC refill fluids or choking on an EC flavor cartridge (Durmowicz, 2014). Additionally, EC products and refill fluids provide a new readily accessible source of nicotine that can be used for intentional poisoning.

Although the case reports do not identify specific chemicals causing health outcomes, the reported systemic effects (*e.g.* infantile necrotizing colitis, RCVS, ulcerative colitis, atrial fibrillation, acute myocardial infarction) could be caused by nicotine, which has been linked to gastrointestinal, neurological, and vascular disorders (Naik and Cucullo, 2015, Mishra et al., 2015). For instance, nicotine could trigger atrial fibrillation and other abnormal cardiovascular events, such as the acute myocardial infarction in a previously healthy man (Kivrak et al., 2014). Additionally, nicotine has potential vasoconstrictive effects on the brain and can induce inflammation in the lungs that imitates metastatic cancer (Naik and Cucullo, 2015, Mishra et al., 2015, Madsen et al., 2016). EC aerosols are also rich in propylene glycol and glycerin. Although these are usually considered non-toxic (ATDSR, 2011), the amounts being inhaled by EC users are large and may be of concern (Behar et al., 2015, Burstyn, 2014). Propylene glycol can cause allergic and inflammatory reactions and irritate the lungs, skin and eyes (Choi et al., 2011), and all of the respiratory diagnoses included inflammation. Persistent lung inflammation caused by EC aerosols could lead to lung pathogenesis and trigger serious diseases such as chronic obstructive pulmonary disease and fibrosis (Rowell and Tarran, 2015).

The reports concerning relapsed and improved UC are the first case reports linking EC use to these conditions (Lee et al., 2013, Camus et al., 2014, Gillen and Saltzman, 2014). For patients with UC, smoking conventional cigarettes can have beneficial and protective effects (Thomas et al., 1998, Calkins, 1989). The cases with opposite effects in EC users suggest that there are factors other than nicotine consumption that work to improve or aggravate conditions in patients with preexisting conditions. It is also important that ECs vary considerably in their performance and contents (Trtchounian et al., 2010, Williams and Talbot, 2011, Williams et al., 2013, Williams et al., 2014, Williams et al., 2016) and opposite effects, as reported with UC, could be related to the specific products used by the patients and the efficiency of their delivery. In addition, topography differs significantly among EC users (Behar et al., 2014a, Behar et al., 2014b). This may also affect nicotine and propylene glycol delivery, depth of penetration of aerosol into the lungs, and total amount of aerosol exposure, all of which may influence health outcomes (Behar et al., 2014a, Behar et al., 2014b). Individual EC usage (product and liquid nicotine), smoking history, and pre-existing health history need to be monitored and considered carefully to better understand the opposing effects of ECs on users with a history of UC.

While EC are sometimes recommended by physicians to pregnant women as a less harmful alternative than tobacco cigarettes and are perceived as safe to use during pregnancy by pregnant women (Mark et al., 2015, Kahr et al., 2015), our data suggest that pregnant women should approach EC use with caution and be informed of the potential complications that may affect their pregnancy. This idea is further supported by *in vitro* toxicity studies that have reported that embryonic cells are more sensitive to EC refill fluids than differentiated adult cells of the lungs (Bahl et al., 2012). Additionally, studies have shown that EC exert negative prenatal and postnatal effects on lung growth and adult behavior of mice (McGrath-Morrow et al., 2015, Smith et al., 2015).

The two case reports dealing with mechanical injury resulted from spontaneous explosion of the EC battery. Such accidents can be triggered by modification of the EC, overheating, or using an incorrect battery charger (USFA, 2014). EC explosions can result in fires, explosions, severe injuries, burns, and death (Electronic cigarette fires and explosions. Emmitsburg: US F, Durmowicz, 2014). Most explosion related injuries have not appeared in the peer reviewed literature, but a recent report by the 2014 US Fire Administration concisely documents many incidents of exploding EC that have caused injury to users (USFA, 2014). Numerous reports of explosions have appeared in print and news media and lawsuits have been filed to compensate victims of such explosions (LA Times, 2015). The Department of Transportation recently prohibited storage of EC in checked baggage on airplanes to avoid damage and injury due to an explosion (Department of Transportation, 2015).

In summary, EC have their own set of health effects that need to be better characterized and understood. Data from case reports show that EC use can be accompanied by negative and, less frequently, positive health effects. Health was affected by EC use in both adults and children (non-users), as well as adults without pre-existing conditions, adults with mental illness, and a newborn exposed during pregnancy. For nicotine poisonings and injuries due to EC explosion, the number of case reports and range of negative effects are not representative of actual incidents. Many additional reports have appeared in print media and on government and medical websites that collect health data. It is also likely that the systemic effects caused by EC are currently under-reported in peer-reviewed literature. Although most individuals who experienced negative health effects eventually recovered, their risk for recurrence is an important issue for health care professionals to consider.

Finally, a consistent and uniform presentation of data in future case reports dealing with EC is needed. Along with patient demographics and smoking history, previous health history, and positive and negative diagnoses, it would be helpful if case reports included details pertaining to: (1) the frequency of EC use; (2) EC nicotine concentration, brands, and flavors; (3) the time of onset of symptoms after EC use; (4) the conditions that are linked to EC; (5) if symptoms reversed after cessation of EC; and (6) follow-up reports on patients.

## Conflict of interest

None.

## Financial disclosure

No financial disclosures were reported by the authors of this paper.

## Figures and Tables

**Table 1:**
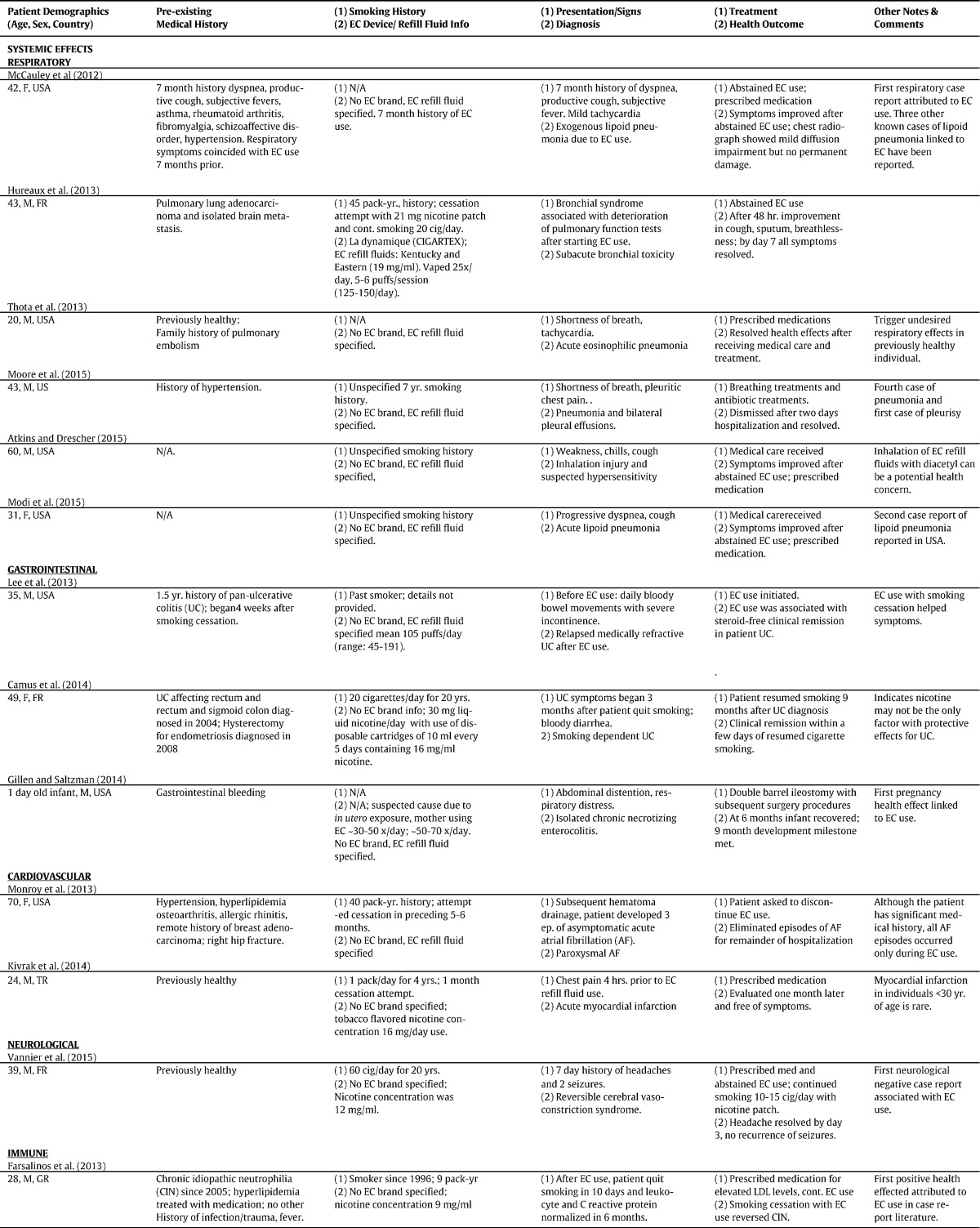

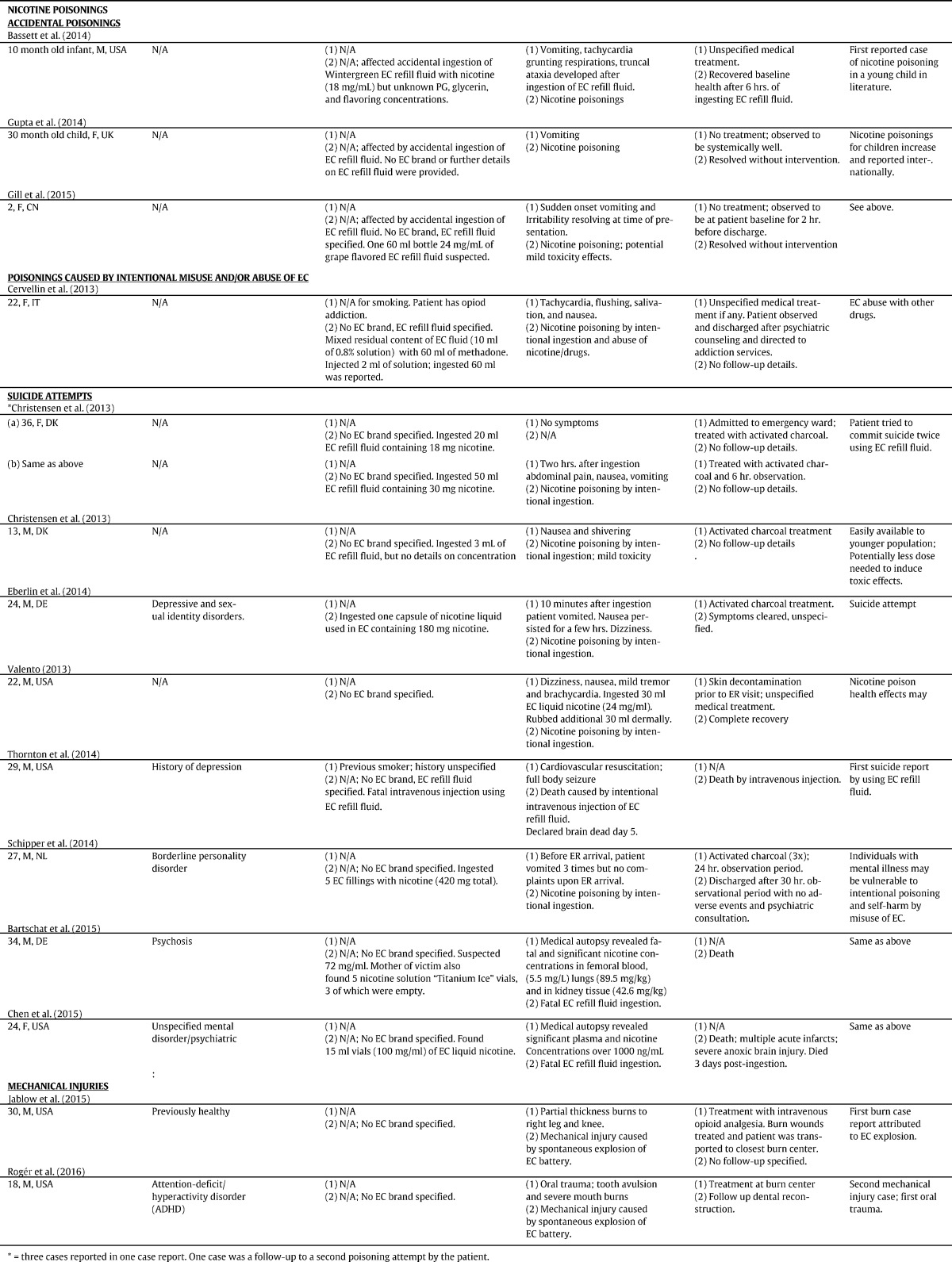
Case reports involving EC health effects.
